# Acute Hypophagia and Changes in c-Fos Immunoreactivity in Adolescent Rats Treated with Low Doses of Oxytocin and Naltrexone

**DOI:** 10.3390/jcm11010059

**Published:** 2021-12-23

**Authors:** Mitchell A. Head, Laura K. McColl, Anica Klockars, Allen S. Levine, Pawel K. Olszewski

**Affiliations:** 1 Faculty of Science and Engineering, University of Waikato, Hamilton 3214, New Zealand; m.headscience@outlook.com (M.A.H.); Laura.mccoll@waikato.ac.nz (L.K.M.); anica.klockars@waikato.ac.nz (A.K.); 2 Department of Food Science and Nutrition, University of Minnesota, St. Paul, MN 55113, USA; aslevine@umn.edu; 3 Department of Integrative Biology and Physiology, Medical School, University of Minnesota, Minneapolis, MN 55416, USA

**Keywords:** neuropeptides, obesity, satiety

## Abstract

A recent case report has shown that an adjunctive oxytocin + naltrexone (OT + NTX) treatment promoted more robust hypophagia and body weight reduction than OT alone in an adolescent male with hypothalamic obesity after craniopharyngioma resection. Thus far, there has been no basic research in adolescent laboratory animals that would examine whether the benefit of OT + NTX on appetite extends onto adolescent individuals without surgically induced overeating. Thus, here we examined whether low doses of combined OT + NTX acutely affect post-deprivation intake of energy-dense, standard chow; intake of energy-dense and palatable high-fat high-sugar (HFHS) diet; or calorie-dilute, palaTable 10% sucrose solution without deprivation in adolescent male rats. We assessed whether OT + NTX decreases water intake after water deprivation or produces a conditioned taste aversion (CTA). Finally, by using c-Fos immunoreactivity, we determined changes in activity of feeding-related brain areas after OT + NTX. We found that individual subthreshold doses of OT and NTX decreased feeding induced by energy and by palatability. Significant c-Fos changes were noted in the arcuate and dorsomedial hypothalamic nuclei. The hypophagic doses of OT + NTX did not suppress water intake in thirsty rats and did not cause a CTA, which suggests that feeding reduction is not a secondary effect of gastrointestinal discomfort or changes in thirst processing. We conclude that OT + NTX is an effective drug combination to reduce appetite in adolescent male rats.

## 1. Introduction

Approximately one third of American children and adolescents are overweight or obese [1]. Behavioral, dietary, and lifestyle recommendations constitute key treatment approaches; however, considering the high prevalence of obesity at an early age and its negative consequences potentially lasting a lifetime, there is growing interest in developing successful pharmacotherapies for these age groups [2]. In 2017, Hsu and colleagues published exciting results of a case study involving an adolescent male with craniopharyngioma resection-induced hypothalamic obesity and loss-of-control overeating. In that study, the authors initially treated the patient with intranasal OT to in order to lower food intake and body weight [3]. Following 10 weeks of the OT-alone administration, they expanded the treatment by co-administering OT with an opioid receptor antagonist, naltrexone (NTX). They found OT alone to be effective at reducing body weight and overeating, however, the adjunctive OT + NTX therapy promoted more robust changes in appetite and BMI.

The benefit of the combined OT + NTX on appetite is reflected by how these molecules affect energy intake. Human and laboratory animal studies strongly link opioids with eating for reward, and thus with consumption driven by pleasant taste regardless of the actual energy needs of the organism. Yeomans and Gray reported that blocking opioid receptors with 50 mg NTX decreased meal size in adult male subjects and it diminished the rated pleasantness of the food and, correspondingly, the speed of consumption [4]. In another human trial, pleasantness of sweet, high-fat, and high-protein palatable foods has been found to be significantly affected by NTX [5]. In line with that, Arbisi and colleagues showed that while NTX did not change taste detection or recognition, it did reduce the pleasantness of sucrose solutions [6]. Animal experiments reveal that opioid receptor antagonists, including NTX, suppress feeding. Importantly, they are particularly effective in reducing consumption driven by palatability but not by energy needs. Operant studies indicate that opioid ligands do not change a perceived level of hunger [7]. Only high doses of naloxone decrease scheduled deprivation-induced standard chow intake, whereas intake of sugary pellets is readily suppressed by low doses of the opioid antagonist, and that is observed even when food is available for just 30 min [8]. Morales et al. have reported that NTX decreases sugar solution intake in the mouse model of binging [9]. Finally, naloxone injected in animals habitually consuming sugar produces withdrawal-like effects, including head shakes, teeth chattering and tremor, which reflects the role of opioids in neural processing of feeding reward [10].

On the other hand, OT appears to be particularly effective in strengthening satiation, and thus ‘homeostatic’ rather than reward-related facets of eating behavior. Heightened activation of the OT system coincides with stomach distension and elevated plasma osmolality, i.e., the parameters that typically lead to inhibition of food intake [11]. In animal models, central and peripheral OT injections decrease feeding [12,13,14]. OT limits meal duration and lowers intake of standard chow [15]. Unlike opioid receptor antagonists, OT’s ability to affect feeding for reward appears more limited. The flavor, calorie density, macronutrient composition, and even social reward associated with presentation of a meal, modify responsiveness to OT [16,17,18,19,20]. In human studies, intranasal OT in obese and normal-weight subjects acutely decreases food intake [3,21,22,23,24]. A potential link with some aspects of eating for reward has been suggested, though the details remain to be elucidated, as some authors found preferential effects of OT on ingestion of either sweet [22,23,25], fat [21], or salty [23] diets.

By combining the complementary actions of the OT (primarily homeostatic) and NTX (primarily reward-related) on feeding, one might intuitively anticipate some level of synergy. The case study by Hsu et al. writes well into this hypothesis, though considering that the treatment was done in an adolescent in whom overeating developed as a result of the craniopharyngioma resection, there is a reasonable doubt that outcomes might differ in adolescents whose overeating is not driven by the prior surgical damage to the hypothalamus. We have recently reported that adult animals subjected to the combined OT + NTX treatment indeed eat less of the high-fat high-sugar diet presented right after the injection, but in that case, the experiments were performed in adult rather than adolescent rats [26]. Importantly, adolescents show heightened sensitivity to rewarding stimuli, including food [27]. This gap in knowledge calls for assessing the effectiveness of the OT + NTX combination on feeding in adolescent animals eating either standard or palatable foods.

In the current project focused on feeding behavior in adolescent rats, we determined whether low doses of combined OT and NTX acutely affect (a) post-deprivation intake of energy-dense, standard (‘bland’) chow, (b) intake of energy-dense and palatable high-fat high-sugar (HFHS) chow in nondeprived animals, or (c) ingestion of calorie-dilute, palatable 10% sugar water in nondeprived animals. We assessed whether the effective OT + NTX combination decreases water intake after overnight water deprivation and whether OT + NTX produces a conditioned taste aversion (CTA), which would indicate that hypophagia might stem from sickness/malaise. Finally, by using c-Fos immunoreactivity, we determined changes in activity of feeding-related brain areas in response to the OT + NTX treatment. Similar to the previously published studies on OT and NTX in adult rats [26] in which a synergistic effect of the two molecules was anticipated, we used the doses of OT and NTX that—if administered alone—were subthreshold. This is because the ultimate goal of combination treatments is to use very low drug doses (well below the minimum effective doses in single drug treatment regimens) to minimize other (undesirable) effects of each compound.

## 2. Materials and Methods

### 2.1. Animals and Drugs

Adolescent male Sprague–Dawley rats (postnatal days 28–40, similar to previously reported [28] were housed individually in Plexiglas cages with wire tops in a temperature-controlled (22 °C) animal room with a 12:12 light:dark cycle (lights on at 07:00)). Standard laboratory chow pellets (Sharpes, New Zealand; energy density: 3.6 kcal/g) and tap water were available ad libitum unless noted otherwise. Animals were treated in accordance with the National Institute of Health Guide for the Care and Use of Laboratory Animals. The University of Waikato Animal Ethics Committee approved all procedures described herein.

Animals were accustomed to handling and to receiving intraperitoneal (IP) injections. NTX (Abcam, Cambridge, UK) and OT (Sigma, St. Louis, MO, USA) were dissolved in isotonic saline just prior to use.

### 2.2. Feeding Studies

#### 2.2.1. Establishing below-Threshold Dose for Individually Administered OT in the HFHS Meal Paradigm in Nondeprived Rats

As shown in [15], IP and subcutaneous OT decreases various aspects of consumption at a standard dose of 1 mg/kg body weight (unlike opioid receptor antagonists whose effective doses are greatly dependent on palatability of food (e.g., [7,8])). In order to confirm that indeed the 1 mg/kg OT dose is sufficient to decrease consumption in our cohort of adolescent animals, we used the palatable high-fat high-sugar (HFHS) diet paradigm previously published in relation to OT’s effect on feeding [26].

Rats were pre-exposed to the HFHS chow to prevent neophobia 4 days prior to the study (2 h). On the experimental day, standard chow was removed from cages at 10:00 (access to water was uninterrupted), and 15 min later the rats were injected IP with vehicle (0.9% saline) or OT at 0.1 mg/kg, 0.3 mg/kg, or 1 mg/kg b. wt. (*n* = 8–9/group; 31 total). Immediately after the treatment, they were given the HFHS diet and the consumption was measured 2 h later. In this paradigm, the HFHS chow consumption occurs without any prior energy deprivation, and thus the motivation to initiate feeding is driven primarily by hedonics. Data from the OT-injected groups were compared with the controls with ANOVA followed by Dunnett’s test. Differences were considered significant for *p* ≤ 0.05. Effect sizes were determined with Cohen’s d and Hedges’ g values (here and thereafter, d is used for same-size group comparisons and g for groups that differ in size). By convention, effect sizes exceeding 0.8 are considered large.

**Figure 1:**
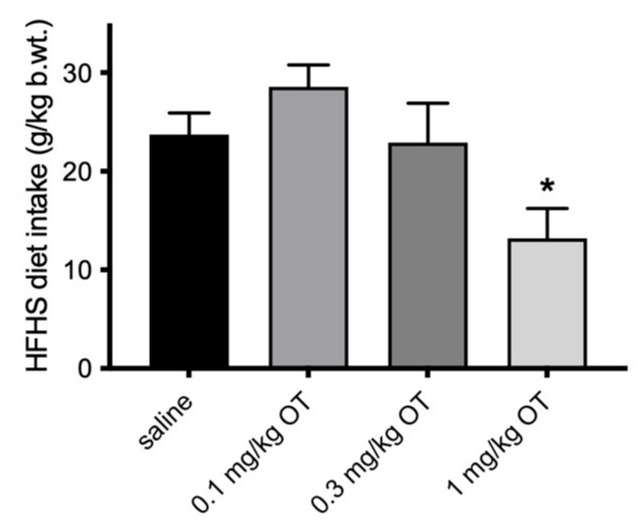
Effect of IP saline (control), 0.3 mg/kg OT, 0.3 mg/kg OT, or 1 mg/kg OT on 2-h episodic intake of energy-dense and palatable HFHS chow in nondeprived rats. * *p* < 0.05 (significantly different from saline controls).

The experiment confirmed that, indeed, 1 mg/kg OT was the minimum dose to reduce feeding (Figure 1) and, therefore, in all subsequent experiments, as a subthreshold dose of OT, we used 0.1 mg/kg OT (and thus, 10 times less than the lowest effective dose).

#### 2.2.2. Episodic Intake of the HFHS Diet in Nondeprived Rats after NTX or after Co-administration of OT and NTX

As the effect of opioid receptor antagonists on feeding is greatly dependent on palatability and energy density of a diet, in this and all subsequent food intake studies, we first determined the effect of various doses of IP NTX on HFHS intake, and then used these doses in combination with the 0.1 mg/kg OT (and thus, with the lowest effective dose established in Section 2.2.1).

We used the same HFHS feeding paradigm as described above in Section 2.2.1., i.e., on the experimental day, standard chow was removed at 10:00, and rats (pre-exposed to HFHS 4 days earlier) were given IP saline or NTX at 1 mg/kg, 3 mg/kg, or 10 mg/kg (*n* = 8–9/group; 34 total). Immediately after the treatment, they were given the HFHS diet and the consumption was measured 2 h later. It should be noted that the 2 h feeding period used in this and subsequent experiments was chosen based on the effective 2 h timeframe for the OT + NTX treatment published in the earlier report that focused on feeding responses in adult animals (hypophagia was found to be a short-lived phenomenon in that previous study) [26].

In the OT-NTX co-administration study, animals were injected either with vehicle (saline), 0.1 mg/kg OT, 0.1 mg/kg OT + 1 mg/kg NTX, 0.1 mg/kg OT + 3 mg/kg NTX, or 0.1. mg/kg OT + 10 mg/kg NTX (*n* = 8–9/group; 42 total including the 34 used 4 days earlier for the NTX dose-response randomly assigned to treatment groups).

Data from the drug-injected groups were compared with the controls with ANOVA followed by Dunnett’s test. Differences were considered significant for *p* ≤ 0.05. Effect sizes were determined with Cohen’s d and Hedges’ g values

#### 2.2.3. Episodic Intake of 10% Sucrose Solution in Nondeprived Rats after NTX or after Co-administration of OT and NTX

In the NTX-alone dose-response study, on the experimental day, standard chow and water were removed at 10:00, and rats (pre-exposed to 10% sugar solution 4 days earlier) were given IP saline or NTX at 0.1 mg/kg, 0.3 mg/kg, or 1 mg/kg (*n* = 6/group; 24 total). Immediately after the treatment, they were given pre-weighed bottles containing the sweet solution and the consumption was measured by weighing the bottles after 2 h.

In the OT-NTX co-administration study, animals were injected either with vehicle (saline), 0.1 mg/kg OT, 0.1 mg/kg OT + 0.1 mg/kg NTX, 0.1 mg/kg OT + 0.3 mg/kg NTX, or 0.1. mg/kg OT + 1 mg/kg NTX (*n* = 6–9/group; 39 total including the 24 used 5 days earlier for the NTX dose-response randomly assigned to treatment groups).

Data from the drug-injected groups were compared with the controls with ANOVA followed by Dunnett’s test. Differences were considered significant for *p* ≤ 0.05. Effect sizes were determined with Cohen’s d and Hedges’ g values.

#### 2.2.4. Deprivation-Induced Standard Chow Intake after NTX or after Co-administration of OT and NTX

We examined whether NTX or OT + NTX affect feeding induced by energy needs, i.e., intake of standard chow following overnight food deprivation. Food was removed at 17:00 on the day preceding drug administration; water was available at all times.

In the NTX-alone dose-response study, pre-weighed chow was returned to cages at 10:00. Just before re-feeding, rats were given IP saline or NTX at 1 mg/kg, 3 mg/kg, or 10 mg/kg (*n* = 6/group; 24 total). Food intake was measured after 2 h.

In the OT-NTX co-administration study, animals were injected either with vehicle (saline), 0.1 mg/kg OT, 0.1 mg/kg OT + 1 mg/kg NTX, 0.1 mg/kg OT + 3 mg/kg NTX, or 0.1. mg/kg OT + 10 mg/kg NTX (*n* = 6/group; 30 total including the 24 used 4 days earlier for the NTX dose-response randomly assigned to treatment groups).

Data from the drug-injected groups were compared with the controls with ANOVA followed by Dunnett’s test. Differences were considered significant for *p* ≤ 0.05. Effect sizes were determined with Cohen’s d and Hedges’ g values

### 2.3. Water Intake and Aversion Assessments

Considering the anorexigenic consequences of the OT-NTX treatment observed in our feeding studies, we also wished to assess whether this hypophagia may be unrelated to feeding itself, but it rather may be a residual consequence of either a changed thirst sensation or a gastrointestinal discomfort induced by the treatment. We therefore examined whether 0.1 mg/kg OT + 3 mg/kg NTX (the combination of doses sufficient to decrease consumption in all of the three feeding paradigms investigated by us) reduces water drinking behavior after water deprivation (thirst) or generates a conditioned taste aversion, i.e., avoidance of a novel tastant whose intake was associated with sickness/malaise produced by the drug treatment.

#### 2.3.1. Water Intake after Overnight Water Deprivation

Water was removed at 18:00 on the day preceding the injection; standard chow was available during the hours of water deprivation. On the experimental day, the rats were injected IP with vehicle (0.9% saline), OT at 0.1 mg/kg, 3 mg/kg NTX, or 0.1 mg/kg OT + 3 mg/kg NTX (*n* = 7–8/group; 29 total). Immediately after the treatment, pre-weighed water bottles were returned to cages and water consumption was measured 2 h later. During the 2 h of water intake measurement, standard chow was taken away. The removal of standard chow was done in light of the results of the feeding studies in which OT + NTX reduced chow consumption and the fact that intake of solid food by itself affects the amount of consumed water. Data were compared with the controls with ANOVA followed by Dunnett’s test. Differences were considered significant for *p* ≤ 0.05.

#### 2.3.2. Conditioned Taste Aversion

Rats were deprived of water at 18:00. On the next day at 10:00, they were given a bottle containing a novel 0.1% saccharin solution instead of water. After 1 h of drinking, they were injected IP with vehicle (saline), 23.9 mg/kg LiCl (known to induce a CTA at this dose—a positive control for CTA), 0.1 mg/kg OT, 3 mg/kg NTX, or 0.1 mg/kg OT + 3 mg/kg NTX (*n* = 6–7/group; 31 total). During the next 2 days after injections, animals had unrestricted access to water and chow. They were subsequently deprived of water again and, on the next day (at 10:00), a standard two-bottle (water vs. 0.1% saccharin) preference test lasting 1 h was used to assess acquisition of CTA to saccharin. Chow was present in cages at all times with the exception of the two bottle test and the initial 1 h novel saccharin solution presentation.

Bottles were weighed before and after the CTA test. The amount of consumed 0.1% saccharin solution was expressed as the percentage of total fluid intake during the two-bottle test. The data were analyzed with ANOVA followed by Dunnett’s test (significant for *p* ≤ 0.05). Effect sizes were determined with Cohen’s d and Hedges’ g values.

### 2.4. c-Fos Immunohistochemistry

Rats were divided into four groups based on the injectant (*n* = 9/group; 36 total): each received an IP injection of either isotonic saline, 0.1 mg/kg OT, 3 mg/kg NTX, or 0.1 mg/kg OT + 3 mg/kg NTX. Food and water were taken away at the time of drug administration. An hour after drug administration, animals were deeply anesthetized with urethane (35% dissolved in 0.9% saline, IP) and perfused through the aorta with 50 mL of saline (at room temperature) followed by 500 mL of 4% paraformaldehyde (PFA) in 0.1 phosphate buffer (pH 7.4). Brains were excised and post-fixed overnight in PFA at 4 °C. 60 μm-thick coronal sections were cut with a vibratome (Leica, Frankfurt, Germany) and later processed as free-floating sections for standard single antigen c-Fos immunostaining.

Sections were rinsed in 50 nM TBS (pH 7.4–7.6) and then pretreated for 10 min in 3% H_2_O_2_ and 10% methanol (in TBS). After rinsing in TBS (4 × 10 min) they were incubated overnight at 4◦C in the polyclonal rabbit-anti-Fos antibody (1:4000; Synaptic Systems, Murarrie, Australia) washed in TBS, and incubated at room temperature for 1 h in the goat-anti-rabbit antibody (1:400; Vector Laboratories, Burlingame, CA, USA). After four washes in TBS, sections were incubated for 1 h with the avidin–biotin peroxidase complex (1:800; Elite Kit, Vector Laboratories, Burlingame, CA, USA). The vehicle for all incubations was a TBS-based solution of 0.25% gelatin and 0.5% Triton X-100. The peroxidase in the sections was visualized with 0.05% diaminobenzidine (DAB), 0.01% H_2_O_2_, and 0.3% nickel sulfate (10 min) in TBS. Sections were rinsed four times in TBS to stop the reaction, mounted onto gelatin-coated slides, air-dried, dehydrated in alcohol, soaked in xylene (Merck KGaA, Darmstadt, Germany), and embedded in Entellan (Merck KGaA, Darmstadt, Germany). The number of Fos-immunoreactive nuclear profiles per 1 mm^2^ was counted bilaterally for each neuroanatomical area of interest using ImageJ software. The boundaries were determined according to the Paxinos and Watson brain atlas on 2–4 sections per animal. Images captured via a CCD camera attached to a Nikon Eclipse 400 microscope were analyzed using Nikon NIS Elements image software. The following regions were included in the analysis (anterior-posterior ranges of bregma levels are shown in the parentheses): AcbC—nucleus accumbens core (1.28–0.96); AcbS—nucleus accumbens shell (1.28–0.96); ARC—arcuate nucleus (−2.16 to −2.52); BLA—basolateral nucleus of the amygdala (−2.64 to −2.92); CEA—central nucleus of the amygdala (−2.64 to −2.92); DMH—hypothalamic dorsomedial nucleus (−3.00 to −3.24); DMNV—dorsal motor nucleus of the vagus (−13.76 to −14.16); NTS—nucleus of the solitary tract (−13.76 to −14.16); PVN—hypothalamic paraventricular nucleus (−1.56 to −1.92); VMH—ventromedial nucleus (−3.00 to −3.24); LH—lateral hypothalamus (−1.20 to −1.44); BNST—bed nucleus of the stria terminalis (−0.24 to −0.48); and VTA—ventral tegmental area (−6.72 to −6.84).

Group means of densities of Fos-immunoreactive nuclei were compared with ANOVA followed by Dunnett’s test. Values were considered significantly different for *p* ≤ 0.05. Effect sizes were determined with Cohen’s d and Hedges’ g values.

## 3. Results

**Figure 2:**
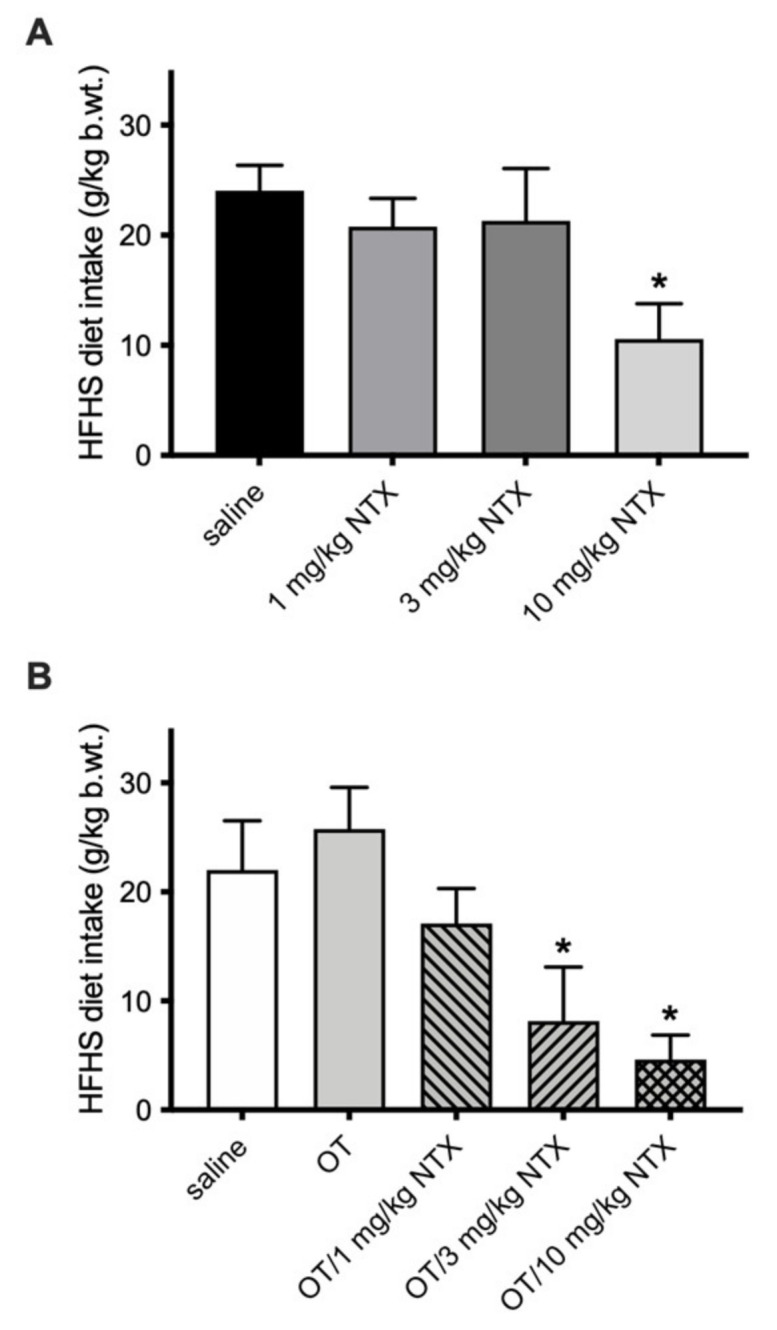
Episodic 2 h HFHS diet intake in nondeprived rats injected IP with: (**A**) saline, 1 mg/kg NTX, 3 mg/kg NTX, or 10 mg/kg NTX; or (**B**) saline, 0.1 mg/kg OT, 0.1 mg/kg OT + 1 mg/kg NTX, 0.1 mg/kg OT + 3 mg/kg NTX, or 0.1 mg/kg OT + 10 mg/kg NTX. * −*p* < 0.05 (significantly different from saline controls).

Administration of 1 mg/kg OT in non-deprived animals given episodic access to the HFHS produced a decrease in consumption (F(3, 27) = 4.34; *p* = 0.044; d = 1.46), whereas lower doses of the peptide were ineffective in reducing food intake (Figure 1). In the same HFHS scenario, NTX was effective at 10 mg/kg: the animals consumed approximately 40% of the saline control group’s amount (Figure 2A; F(3, 30) = 3.01; *p* = 0.019; d = 1.70). Importantly, when the subthreshold 0.1 mg/kg dose of OT was combined with 10 mg/kg NTX, the rats ate about 20% of the amount ingested by controls (F(3, 30) = 4.18; *p* = 0.0103; g = 1.61), and the OT + 3 mg/kg NTX showed significant decrease (*p* = 0.047; g = 1.04; Figure 2B).

**Figure 3:**
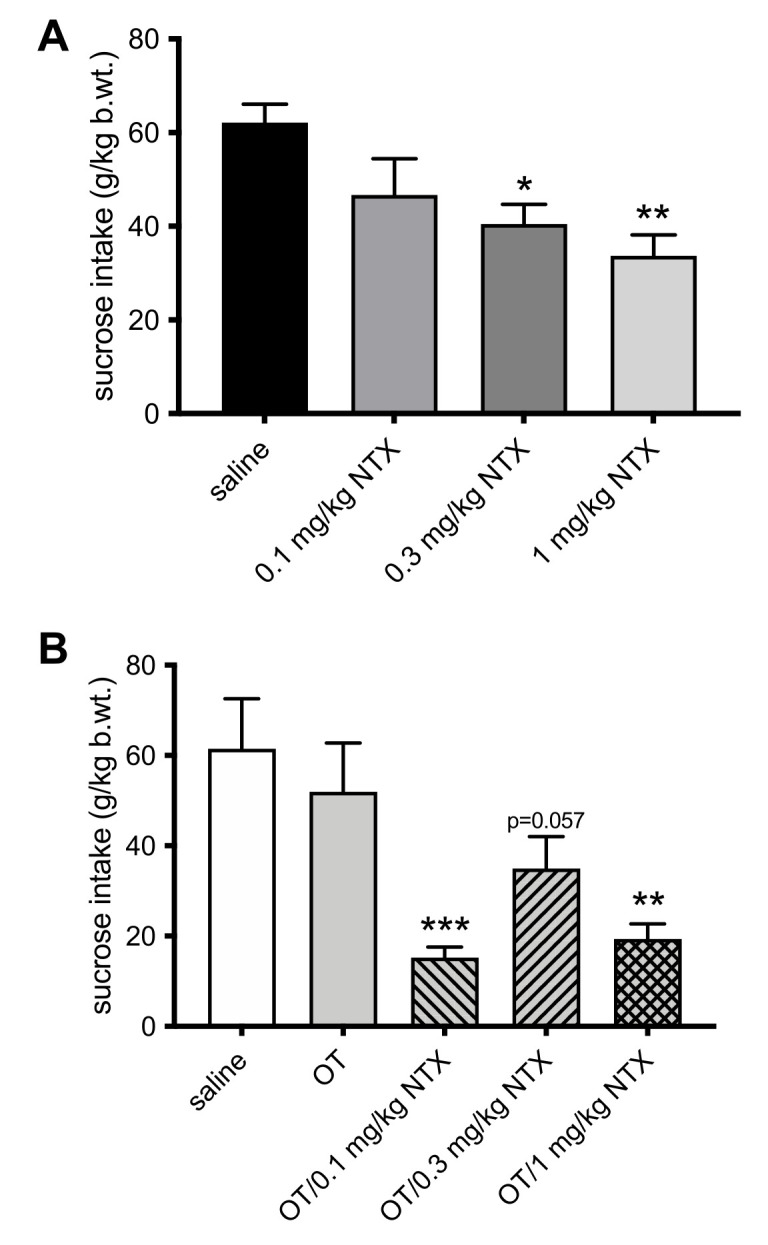
Episodic 2 h 10% sucrose solution diet intake in nondeprived rats injected IP with: (**A**) saline, 0.1 mg/kg NTX, 0.3 mg/kg NTX, or 1 mg/kg NTX; or (**B**) saline, 0.1 mg/kg OT, 0.1 mg/kg OT + 0.1 mg/kg NTX, 0.1 mg/kg OT + 0.3 mg/kg NTX, or 0.1 mg/kg OT + 1 mg/kg NTX. * −*p* < 0.05; ** −*p* < 0.01; *** −*p* < 0.001 (significantly different from saline controls).

Naltrexone alone already at 0.3 mg/kg (F(3,20) = 5.25; *p* = 0.024); d = 2.10) and 1 mg/kg (*p* = 0.003; d = 2.76) decreased sucrose solution intake in nondeprived rats (Figure 3A). While 0.1 mg/kg OT alone was ineffective, it suppressed sucrose consumption when given in combination with 0.1 mg/kg NTX (F(3, 26) = 8.86; *p* < 0.001; g = 1.92) and 1 mg/kg NTX (*p* = 0.002; g = 1.58) (Figure 3B). It should be noted that the OT−0.3 mg/kg NTX combination only approached significance (*p* = 0.057) though variability in response to the treatment was higher (coefficient of variation of 49.2) here than in other groups (OT + 0.1 mg/kg NTX: 46.5; OT + 1 mg/kg NTX: 41.8).

**Figure 4:**
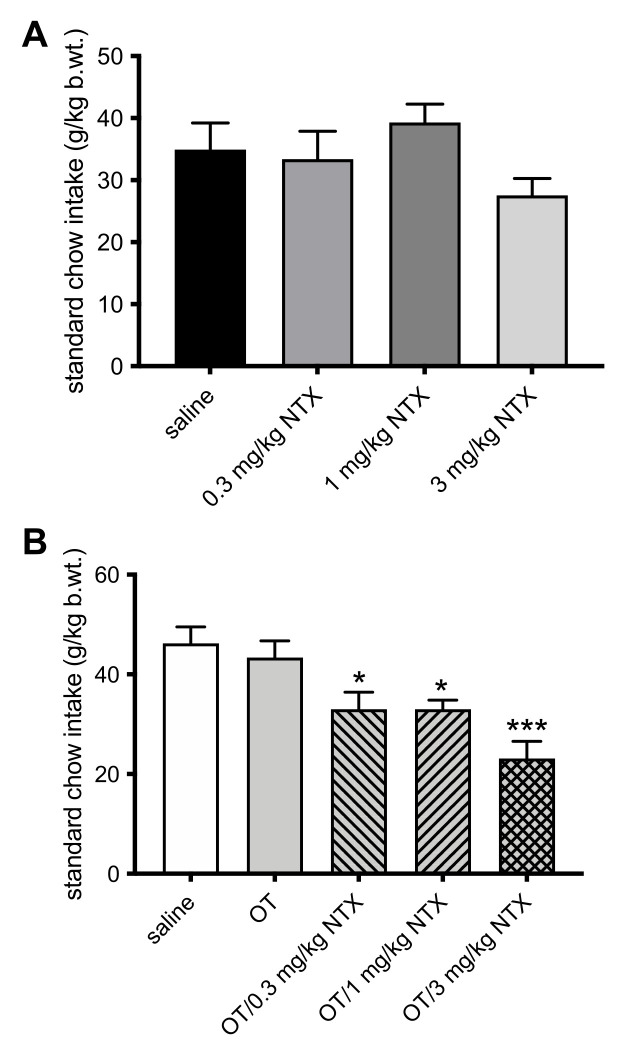
Overnight deprivation-induced standard chow intake (2 h meal) in rats injected IP with: (**A**) saline, 0.3 mg/kg NTX, 1 mg/kg NTX, or 3 mg/kg NTX; or (**B**) saline, 0.1 mg/kg OT, 0.1 mg/kg OT + 0.3 mg/kg NTX, 0.1 mg/kg OT + 1 mg/kg NTX, or 0.1 mg/kg OT + 3 mg/kg NTX. * −*p* < 0.05; *** −*p* < 0.001 (significantly different from saline controls).

Finally, NTX alone did not suppress deprivation-induced intake of standard chow (Figure 4A). When given in combination with the subthreshold 0.1 mg/kg dose of OT, NTX at 0.3 mg/kg (F(3, 20) = 9.64; *p* = 0.016; d = 1.61), 1 mg/kg (*p* = 0.017; d = 2.04), and 3 mg/kg (*p* < 0.001; d = 2.81) reduced feeding (Figure 4B).

**Figure 5:**
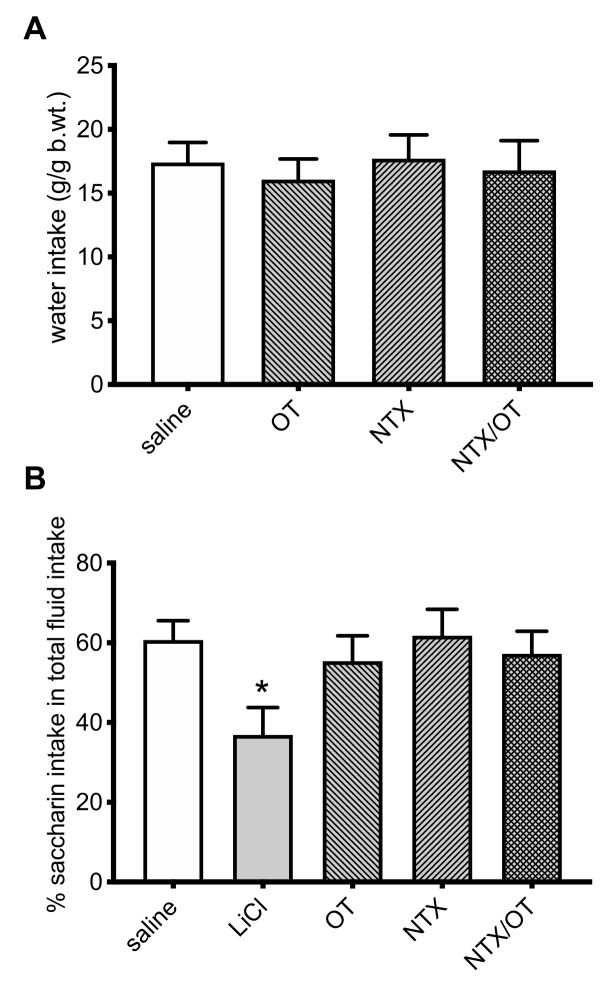
Effect of IP saline (control), 0.1 mg/kg OT, 3 mg/kg NTX, or 0.1 mg/kg OT + 3 mg/kg NTX on: (**A**) 2 h intake of water after overnight water deprivation; or (**B**) acquisition of a conditioned taste aversion (CTA) to a novel 0.1% saccharin solution (LiCl-treated animals served as a positive control for a CTA). * −*p* < 0.05 (significantly different from saline controls).

The OT-NTX treatment did not affect drinking behavior in animals given water following a period of water deprivation (Figure 5A) nor did it cause a development of a conditioned taste aversion to the novel saccharin solution (whereas this avoidance was detected in LiCl-treated rats that served as a positive control of aversion: *p* = 0.029; g = 1.61) (Figure 5B).

**Figure 6:**
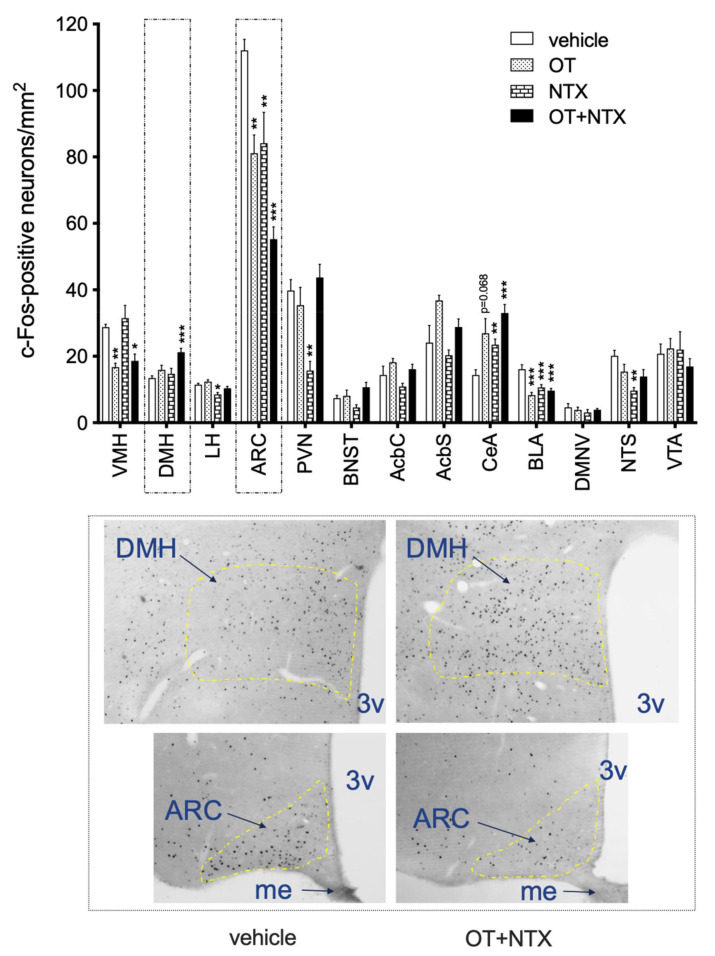
c-Fos immunoreactivity in feeding-related brain areas in rats injected IP with saline, 0.1 mg/kg OT, 3 mg/kg NTX, or 0.1 mg/kg OT + 3 mg/kg NTX. * −*p* < 0.05; ** −*p* < 0.01; *** −*p* < 0.001 (significantly different from saline controls). Photomicrographs depict the dorsomedial (DMH) and arcuate (ARC) nuclei in animals treated with vehicle (saline) and OT + NTX. 3v—third ventricle, me—median eminence.

Among all the brain sites analyzed for c-Fos, the DMH was the only one that showed no change in response to either NTX or OT alone but had a significantly higher c-Fos immunoreactivity in the OT + NTX group (Figure 6; F(3, 32) = 7.22; *p* < 0.001). In the ARC, OT (F(3, 30) = 14.27; *p* = 0.004; g = 2.31) and NTX (*p* = 0.007; d = 1.33) decreased c-Fos expression, the effect was most pronounced with the OT + NTX treatment (*p* < 001; g = 5.59). In addition, all three treatments caused a decrease in Fos in the BLA (F(3, 32) = 13.36; *p* < 0.001 for each; NTX: d = 1.64; OT: d = 2.35; OT + NTX: d = 2.03), and an increase in the CeA (though in the case of OT, it was approaching significance at *p* = 0.068, whereas for NTX *p* = 0.009, d = 0.92, and OT + NTX *p* = 0.001, d = 2.95; F(3,32) = 7.86). In addition, differences were also found in the VMH (OT F(3, 31) = 10.09, *p* = 0.002, d = 3.91, and OT + NTX *p* = 0.013; g = 2.31), LH (NTX F(3, 32) = 9.97, *p* = 0.001, d = 2.1), PVN (NTX F(3, 29) = 7.16, *p* = 0.002, d = 2.83), and NTS (NTX F(3, 28) = 5.45, *p* = 0.0013, g = 2.56) (Figure 6).

## 4. Discussion

We eat for a variety of reasons. While the primary one is to acquire energy to maintain basic functioning of the organism, we also eat for pleasure, to alleviate stress, and to facilitate social interactions, to name a few.

The complexity of eating behavior is paralleled by the plethora of neural and neuroendocrine mechanisms that regulate appetite, each of them differently affecting various facets of feeding (e.g., neuropeptide Y: inducing mainly hunger and dopamine: mainly reward) [29]. Considering this complexity, it is not surprising that the search for pharmacological treatments to reduce overeating has been broadened by incorporating not just a single-molecule approach, but by combining molecules whose unique effects on food intake might yield a beneficial and synergistic change in appetite. To date, Contrave (bupoprion + NTX) and Qsymia (phentermine + topiramate) have gained FDA approval [30].

The choice for pharmacotherapies of obesity and overeating in adolescents is more limited than in adults [31], with liraglutide, orlistat, and phentermine being available. No combination therapy has been approved thus far and the search for effective drug combinations is ongoing. The lag in adolescent therapy development stems from the scarcity of data from human trials and from the fact that even in basic research studies utilizing animal models, the majority of research is conducted in adult organisms.

The case study by Hsu et al. [3] reporting the beneficial effect of the OT + NTX on appetite and body weight in an adolescent patient with hypothalamic obesity induced by craniopharyngioma resection was a welcome discovery showing a new avenue of research toward combination pharmacotherapy for young individuals. The current set of experiments utilizing adolescent animals adds to this initial evidence by showing the effectiveness of OT + NTX in reducing episodic (meal) consumption in young rats.

It is important to note that the OT + NTX-induced suppression of consumption occurred in the context of both feeding for energy and feeding driven primarily by palatability.

In deprivation-driven intake of standard chow, a model which relies on hunger as the main motivator to ingest energy-dense yet “bland” food, NTX alone was—unsurprisingly (in line with previous studies done by us and others [7,26])—ineffective. So was the 0.1 mg/kg OT IP, the dose which—as per this work and previous reports [15]—is well below the 1 mg/kg threshold to produce hypophagia regardless of how feeding is induced. The OT + NTX (with NTX as low as 0.3 mg/kg) generated a significant decrease in standard chow intake post-deprivation. This is an important finding considering that in our recent report utilizing adult rather than adolescent animals, intravenous (IV) OT + NTX did not affect feeding for energy [26]. In line with that, adolescent animals display enhanced sensitivity to deprivation: for example, it has been found that in adolescent rats, energy deprivation abolishes preference for a social stimulus and decreases investigation of social cues, whereas in adult individuals investigation of social stimuli remains intact [32]. Thus, while it is possible that the route of drug delivery in the post-deprivation chow intake in the adult rat study (IV instead of IP) might have affected the outcome, it is quite likely that adolescent individuals show sensitivity to OT + NTX even in the context of energy-driven consumption.

The OT + NTX combination was also effective in another paradigm in which energy-dense (though, in this case, palatable) HFHS chow was given. In this scenario, which capitalizes on the drive to eat induced by combined palatability and energy density of the food, the 1-mg/kg dose of NTX combined with 0.1 mg OT produced hypophagia. A similar decrease in the HFHS diet intake has been reported for adult rats treated with OT + NTX [26].

It should be noted that—unlike in deprived animals—in the HFHS and sucrose solution intake models, NTX alone was effective at reducing consumption. It is consistent with the fact that opioid receptor antagonists more potently suppress ingestion of rewarding foods, whereas agonists more readily promote overconsumption of such tastants (for review, see e.g., [29]). Importantly, NTX doses needed to suppress this type of feeding in our experiments were higher for the NTX-only treatment than when the drug was used in combination with OT. This parallels the previous findings reported for adult animals given HFHS vs. standard chow [26]. Since our current experiments also included the assessment of the effect of OT + NTX on sugar water consumption (and thus, an energy-dilute and highly palatable solution) in nondeprived rats, we posit that the synergistic effect of OT + NTX extends onto feeding driven by palatability.

This finding is also crucial from the broader standpoint of our being able to counteract high susceptibility of young individuals to rewarding stimuli. It has been well documented that juvenile rats relative to adults show a heightened predisposition to ingest high concentrations of ethanol [33] and that adolescent male rats show a pronounced increase in sweetened condensed milk intake, particularly around puberty [34]. Noncaloric saccharin intake in these animals is elevated compared with adults, which indicates that these effects are unrelated to growth-related metabolic needs. Furthermore, adolescent animals subjected to progressive responding operant tasks show greater motivation toward palatable food and other rewards [35,36].

Importantly, our deprivation-induced water intake and CTA experiments indicate that OT + NTX-induced hypophagia are not secondary to either changes in thirst or a feeling of sickness/malaise. Especially the lack of CTA is of significance as opioid receptor blockade with the non-selective antagonist naloxone has been previously shown to potentiate aversive effects of lithium chloride [37], whereas a nociceptin/orphanin FQ receptor antagonist prolonged extinction of a toxin-induced CTA [38].

As with all studies involving OT, one may speculate that—to some extent—hypophagia may be exacerbated by a mild sedating effect of the drug (for example, see [39]). It should be noted, however, that the 0.1 mg/kg OT dose that was used in combination with NTX here is subthreshold to suppress feeding alone (and well below doses widely administered in feeding studies thus far [15]). NTX at doses as high as 10 mg/kg have been found not to promote sedation in rats [40]. It should also be noted that the OT + NTX did not decrease water intake after deprivation and that different doses of NTX combined with OT had to be used to diminish intake of specific tastants, suggesting that the observed hypophagia is at least partially independent from sedation. Nonetheless, it should be emphasized that these results are of pharmacological value and do not presume a physiological relationship.

In order to better understand the impact of the OT + NTX injection on brain activation, we studied cFos in non-deprived animals treated with the drug combination. Since the experiment was performed in rats that were not subjected to food deprivation beforehand and neither were they given access to a specific diet after the injection, the outcome reflects solely the effect of the pharmacological treatment alone in the absence of any dietary or energy status manipulation. The impact of OT + NTX on feeding was reflected by changes in c-Fos immunoreactivity, particularly in the hypothalamic circuit. There was a significant increase in cFos IR in the DMH by the combination treatment, whereas NTX and OT alone were ineffective. Enhanced activity in this region has previously been associated with satiation after re-feeding, whereas presentation of food alone without actual consumption did not lead to changes in DMH c-Fos [41]. On the other hand, in the ARC, we observed the most robust decrease in c-Fos expression by OT + NTX. Interestingly, ARC neurons have been shown to be activated during fasting, e.g., stimulated through the release of ghrelin when the stomach is empty, signaling the need to initiate consumption. Inversely, an anorexigen PYY, released upon stomach distension, has been found to diminish ARC neuronal activation [42]. A similar effect has been reported for PYY analogs [43]. This fits well with the findings of this study that the combination of OT and NTX mirrors ARC activation in a re-fed state. Finally, NTX and OT alone as well as in combination affect activation of the central and basolateral amygdala. Since amygdala activity in response to food cues is exacerbated by energy needs [44], it underscores the effect of each of the compounds on energy-driven consumption. Importantly, the CeA and BLA are typically linked with reward processing. BLA ablation interferes with conditioning and reinforcer devaluation [45], whereas CeA inactivation disrupts learning in response to expectation of reward [46]. BLA encodes emotional processing driven by sensory stimuli [47]. Thus, the concurrent effect on the amygdala reflects the OT + NTX treatment’s effectiveness on consumption motivated by palatability.

One should note that the c-Fos study is not an attempt to make any assumptions regarding the ability of peripheral OT to reach specific central targets. Instead, it only determines whether peripheral OT alone and in combination with NTX engages a different subset of feeding-relevant sites (either in an indirect manner by activating upstream pathways or in a direct fashion), thereby reflecting the differential effect of the treatments on food intake (no effect vs. hypophagia).

It remains to be elucidated whether chronic administration of OT + NTX promotes positive effects on food intake as well as on body weight. Rodent models are suboptimal for determining effects of chronic treatments in adolescents simply due to a very short length of the adolescent developmental phase. However, the earlier study in adult rats receiving a single daily injection found that, unlike OT alone whose effects often wane, the OT + NTX combination maintained its anorexigenic effectiveness over several weeks (though the single injection regimen was insufficient to produce weight loss). It would suggest that chronic administration follow-up studies in adolescents, utilizing animal models more suitable for this phase of life, are warranted.

## 5. Conclusions

We conclude that in adolescent male rats OT + NTX at low doses reduce energy- and palatability-driven consumption without causing aversion or changes in thirst. The hypophagia is paralleled by changes in c-Fos immunoreactivity in key hypothalamic areas: the ARC and DMH.
